# Orbital Osteomyelitis and Periorbital Abscess Due to Coccidioidomycosis Following Trauma

**DOI:** 10.7759/cureus.46586

**Published:** 2023-10-06

**Authors:** Garrick Hayashi, Natalie Pardo, Nurit M Hirsh, Vini Vijayan

**Affiliations:** 1 Department of Pediatrics, Valley Children’s Healthcare, Madera, USA; 2 Department of Pediatrics, California Health Sciences University College of Osteopathic Medicine, Clovis, USA

**Keywords:** coccidioides immitis, endemic fungi, periorbital abscess, orbital osteomyelitis, orbital cellulitis, coccidioidomycosis

## Abstract

Ocular involvement due to *Coccidioides *sp.is extremely rare, and most patients with disseminated coccidioidomycosis present as extrapulmonary or disseminated disease that involves the skin, bone joints, and central nervous system. Here, we describe a 13-year-old previously healthy Black male residing in an area endemic for coccidioidomycosis. The child presented to our hospital with left eye pain, diplopia, and proptosis two weeks after being struck on the left side of the face with a basketball. He was initially presumed to have bacterial orbital cellulitis and was started on empiric antibiotics. Due to severe disease, he underwent surgical drainage and debridement. Fungal stain from the intraoperative specimen showed spherules with endosporulation, and fungal culture revealed *Coccidioides immitis*. Based on this, the child was diagnosed with orbital osteomyelitis and periorbital abscess due to coccidioidomycosis. He was started on intravenous liposomal amphotericin B and fluconazole. Antibiotics were discontinued. He underwent additional investigations to assess for sites of dissemination. His nuclear medicine bone scintigraphy and cerebrospinal fluid studies were negative. A computed tomography (CT) scan of the chest demonstrated multiple small pulmonary nodules. His *Coccidioides *complement fixation(CF) titer was 1:32. The patient completed one month of treatment with liposomal amphotericin B and fluconazole. Our case highlights the need for healthcare professionals to consider coccidioidomycosis when evaluating patients with orbital disease as delays in the diagnosis may result in visual loss and central nervous system involvement. Prompt diagnosis, evaluation, and treatment are crucial to reduce long-term morbidity and mortality.

## Introduction

Coccidioidomycosis, also known as valley fever, is an invasive dimorphic fungal disease caused by fungi of the genus *Coccidioides* (*Coccidioides immitis *and *Coccidioides posadasii*) [1-3]. The fungi are found in dry, arid desert regions and are endemic to the southwest United States including West Texas, New Mexico, Arizona, San Joaquin Valley in California, Northern Mexico, and South and Central America. The annual incidence in these endemic regions is estimated to be 1%-3%, but over the last decade, there has been a drastic rise in the number of cases [2-4]. Disease surveillance of coccidioidomycosis in California from 2000 to 2018 has demonstrated an 800% increase in cases from 2.4 to 18.8 cases per 100,000 persons [2-4]. There has also been a similar increase in the number of cases seen in the pediatric population [5].

Infection typically results from inhalation of airborne arthroconidia, but most infected individuals have asymptomatic or subclinical infections. An estimated 30% of cases develop pulmonary involvement, usually characterized by self-limited community-acquired pneumonia. Less than 0.5%-2% of patients with coccidioidomycosis present with extrapulmonary or disseminated disease [1,6]. Common sites of dissemination include the skin, bone, joints, and lymph nodes, with central nervous system involvement being the most severe. Risk factors for disseminated coccidioidomycosis include impaired cell-mediated immunity such as HIV/AIDs, pregnancy, diabetes, and smoking. Disseminated disease is more common in Hispanics, Filipinos, and African Americans. Epidemiologic data have demonstrated that males are at an increased risk of disseminated infection compared to females [6-9].

Orbital osteomyelitis is an extremely rare and unusual presentation of disseminated coccidioidomycosis [10]. Literature on eye involvement due to coccidioidomycosis is sparse and limited to case reports [10-13]. We report the case of a male residing in an endemic area who presented with orbital cellulitis and osteomyelitis due to coccidioidomycosis following blunt trauma to the eye. He was initially presumed to have bacterial orbital cellulitis and underwent surgical drainage and debridement. Fungal stains from the intraoperative specimen showed spherules with endosporulation, and fungal culture revealed *C. immitis*. The child was diagnosed with orbital osteomyelitis and periorbital abscess due to coccidioidomycosis. To our knowledge, this is the first case describing the occurrence of orbital osteomyelitis due to coccidioidomycosis following trauma.

## Case presentation

A previously healthy 13-year-old African American male presented to the emergency department (ED) with left eye pain, diplopia, and proptosis two weeks after being struck on the left side of the face with a basketball. The child reported nausea, photophobia, and blurry vision of the left eye. He denied fevers, vomiting, headache, or neck stiffness. His mother stated that she had brought him to the ED when he initially sustained the injury. A computed tomography (CT) scan performed at that time showed a left lateral orbital wall fracture, and he was discharged home on analgesics. However, due to the increase in pain and progressive swelling, she brought him back to the ED for further evaluation. His past medical history, surgical history, and family history were non-contributory. The family denied any history of travel or animal exposure. He denied any sick contacts, ingestion of unpasteurized dairy products, or tuberculosis exposures. He had no history of recurrent infections. His immunizations were up to date. The family resided in the San Joaquin Valley of California.

Physical examination revealed an uncomfortable, afebrile child. His temperature was 36.1°C, blood pressure was 128/78 mmHg, heart rate was 84 beats per minute, respiratory rate was 16 breaths per minute, and oxygen saturation rate was 99% on room air. Examination of the eye demonstrated proptosis of the left eye with periorbital erythema and edema. There were no abrasions or disruptions in the overlying skin. He reported limited upward and lateral gaze due to pain. He had normal downward gaze and adduction. The vision was 20/20 bilaterally, and pupils were 4 mm in size, equal, and reactive. The right eye was normal. The remainder of his examination including his neurological examination was within normal limits.

**Figure 1:**
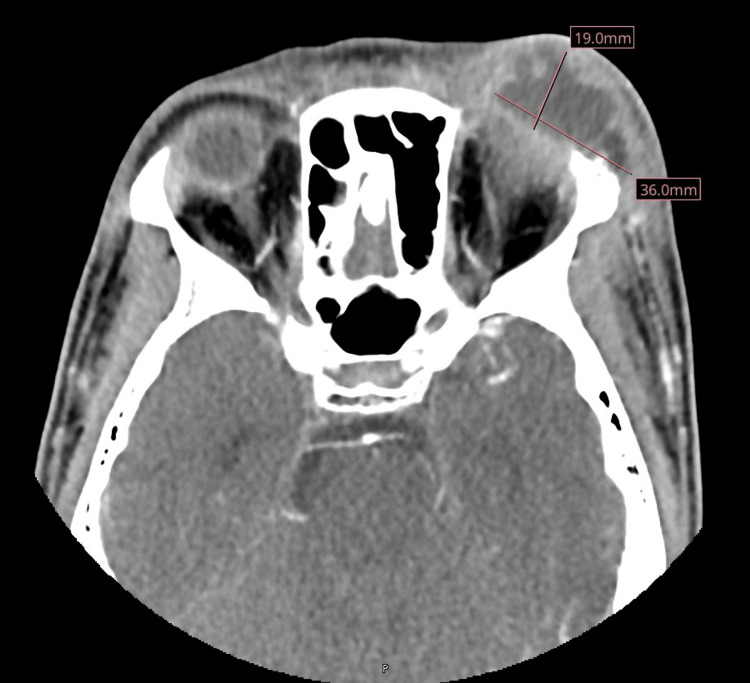
Axial CT of the orbits showing a large left superior lateral periorbital rim-enhancing abscess measuring 4.9 × 3.6 × 1.9 cm and osseous erosions of the superolateral orbital rim CT: computed tomography

**Figure 2:**
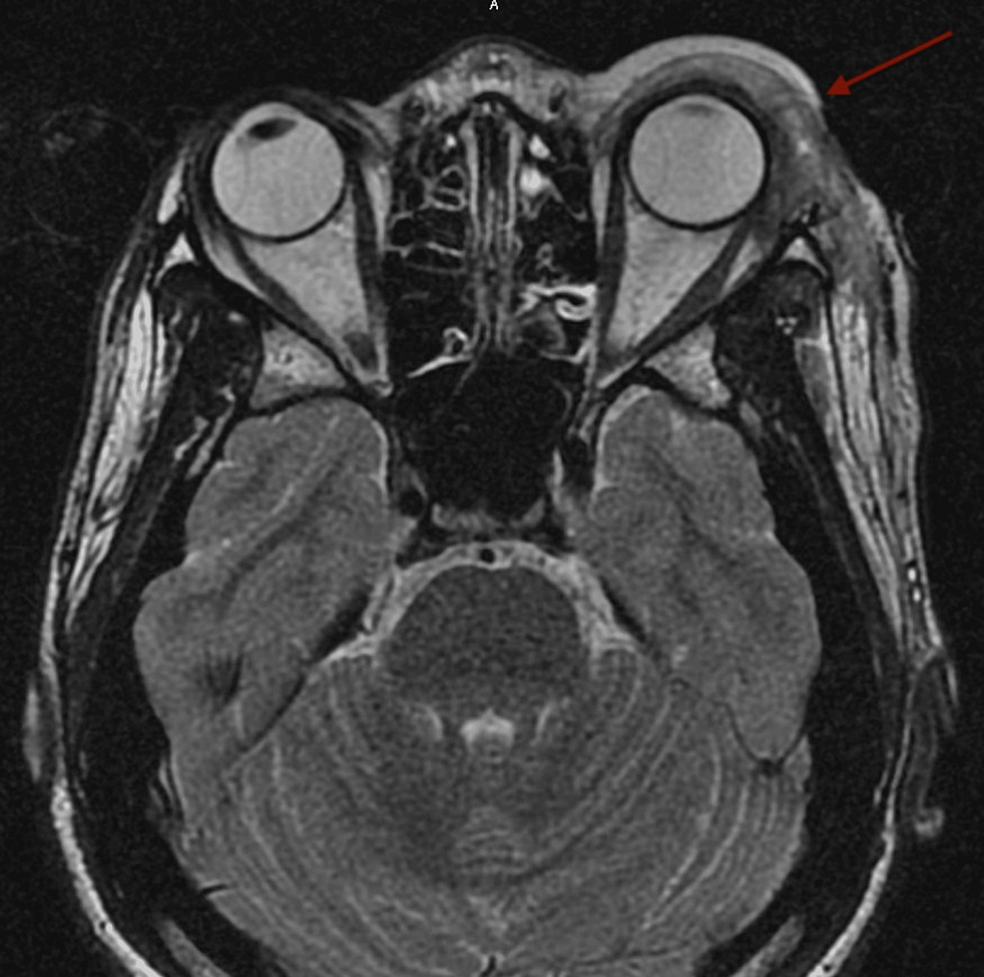
Axial T2 FLAIR magnetic resonance imaging of the orbits showing bony irregularity, edema, and erosions of the superolateral orbital rim consistent with osteomyelitis and post-septal inflammation of the left lateral and superior rectus muscles FLAIR: fluid-attenuated inversion recovery

Initial laboratory evaluation demonstrated normal complete blood counts with a white blood cell count of 7.5 × 10^9^/L with 74% neutrophils, 14% lymphocytes, and 5% monocytes. His hemoglobin was 12.1 gm/dL, and his platelet count was 314 × 10^9^/L. Liver function tests were within normal. His C-reactive protein and erythrocyte sedimentation rate were elevated at 6.3 mg/L and 27 mm/hour, respectively. CT of the orbits showed a large left superior lateral periorbital rim-enhancing abscess measuring 4.9 × 3.6 × 1.9 cm (Figure 1). Magnetic resonance imaging (MRI) of the head and orbits demonstrated erosions of the superolateral orbital rim consistent with osteomyelitis and post-septal inflammation with inflammation of the left lateral and superior rectus muscles (Figure 2). There were no acute intracranial abnormalities detected. The child was admitted for further evaluation and management.

The child was started on an empiric antibiotic regimen comprising ceftriaxone, vancomycin, and metronidazole for presumed bacterial orbital osteomyelitis and periorbital abscess. Ophthalmology was consulted. Examination under anesthesia demonstrated 3+ edema of eyelids and swelling of the lateral aspect of the orbit. He had clear conjunctiva and white sclera, the anterior chamber was deep and clear, the iris was flat, his pupils were round, and the lens and vitreous were also clear. His optic nerve was sharp, the macula was flat, and the retina and vessels were normal. Intraocular pressure was 20 mmHg.

The patient underwent incision and drainage of the abscess and surgical debridement of the left superior orbital rim and frontal bone. There was evidence of fat necrosis of the periorbital tissue, and purulence was expressed. Fungal stain from the intraoperative specimen showed spherules with endosporulation, and fungal culture revealed *C. immitis.* Histopathology of the frontal bone revealed chronic mucosal inflammation with fungal spherules and endospores consistent with coccidioidomycosis. Ziehl-Neelsen and Gram stains were negative for acid-fast bacilli and bacteria, respectively. Based on the cultures and histopathological findings, the diagnosis of orbital osteomyelitis and periorbital abscess due to coccidioidomycosis was made.

The child was started on liposomal amphotericin B at a dose of 5 mg/kg/day and fluconazole at a dose of 12 mg/kg/day. Antibiotics were discontinued. He underwent a workup to assess for additional sites of dissemination. His nuclear medicine bone scintigraphy and cerebrospinal fluid studies were negative. A CT scan of the chest demonstrated multiple small pulmonary nodules. His *Coccidioides *complement fixation (CF) titer was 1:32.

The patient completed one month of treatment with liposomal amphotericin B and fluconazole. A repeat MRI of the orbits showed decreased soft tissue swelling, edema, and enhancement, persistent bony irregularity, and erosion. His visual acuity was 20/20. His repeat *Coccidioides* CF titer decreased to 1:8, and therefore, he was transitioned to oral fluconazole at a dose of 10 mg/kg/day. The patient was discharged on oral fluconazole and close follow-up with ophthalmology and infectious diseases. The child has continued to do well on fluconazole with no signs of relapse at his six-month follow-up outpatient visit.

## Discussion

Ocular manifestations of coccidioidomycosis are classified as intraocular, extraocular, and extraorbital disease. Intraocular manifestations of coccidioidomycosis are generally associated with disseminated coccidioidomycosis and consist of anterior uveitis, chorioretinitis, and endophthalmitis. In contrast, extraocular manifestations of coccidioidomycosis are hypothesized to be hypersensitivity reactions to the coccidioidal antigen and are self-limiting. Manifestations of extraocular coccidioidomycosis include conjunctivitis, scleritis, and keratoconjunctivitis. Extraocular coccidioidomycosis occurs with primary pulmonary infection and is often associated with erythema nodosum. Another rare manifestation of extraocular coccidioidomycosis is lid granulomata. Lid granulomata are often mistaken as a chalazion and are found in disseminated coccidioidomycosis. Extraorbital disease includes optic nerve and cranial nerve abnormalities and is found in patients with cocci involving the central nervous system.

Our case is unique as orbital coccidioidomycosis with osteomyelitis following trauma has not been previously reported. To our knowledge, there is only one case of orbital osteomyelitis with periorbital abscesses reported in the literature [13]. Reed et al. reported the case of a nine-year-old immunocompetent African American male with disseminated coccidioidomycosis who had multiple paravertebral abscesses and extensive osteomyelitis of the T7-T8 vertebrae [13]. During his hospitalization, he developed eye swelling and was incidentally found to have frontal bone superior temporal orbital rim osteomyelitis and periorbital abscess. As this child had extensive disease, his eye involvement was most likely due to hematogenous spread. This case differs as our patient did not have respiratory or other symptoms to suggest hematogenous spread. We suspect that our patient may have developed ocular coccidioidomycosis from direct inoculation following the initial trauma, considering that the child sustained the injury in an area endemic for coccidioidomycosis. Direct inoculation has been previously proposed as a mechanism for orbital coccidioidomycosis by other authors. Hagele et al. reported a case of iridocyclitis that progressed to endophthalmitis and hypothesized that dust entering the eye caused the primary infection [14]. Another possibility is that our patient developed eye involvement due to the contiguous spread of arthroconidia colonizing the paranasal sinuses to the orbit. Ocular disease may have occurred from the reactivation of latent infection, but this often presents with disseminated disease.

There are no definite guidelines for the management of orbital cocci, and the duration of treatment is not known. Guidelines recommended by the Infectious Diseases Society of America (IDSA) recommend fluconazole and liposomal amphotericin B for disseminated coccidioidomycosis, and the treatment can be months to lifelong [15]. Our patient was treated with liposomal amphotericin for one month and subsequently transitioned to oral fluconazole once clinical, serological, and radiographic improvement was achieved with the plan to continue long-term antifungal therapy with clinical and serological monitoring as an outpatient.

## Conclusions

This case underscores the need for healthcare professionals to consider coccidioidomycosis when evaluating patients with orbital disease. In our patient, immediate surgical intervention, microbiological diagnosis, and prompt initiation of antifungal therapy were critical for diagnostic and therapeutic purposes and allowed for a favorable outcome for our patient. Given the rise in cases of coccidioidomycosis, physicians should consider the diagnosis of orbital coccidioidomycosis, when treating children who reside in or have traveled to an area endemic for coccidioidomycosis. Delays in the diagnosis may result in the progression of disease, visual loss, and central nervous system involvement. Prompt diagnosis, evaluation, and treatment are essential to reduce long-term morbidity and mortality. Further studies are needed to establish definitive duration of treatment and prognosis.
